# CCA-addition in the cold: Structural characterization of the psychrophilic CCA-adding enzyme from the permafrost bacterium *Planococcus halocryophilus*

**DOI:** 10.1016/j.csbj.2021.10.018

**Published:** 2021-10-21

**Authors:** Raphaël de Wijn, Kévin Rollet, Felix G.M. Ernst, Karolin Wellner, Heike Betat, Mario Mörl, Claude Sauter

**Affiliations:** a Architecture et Réactivité de l’ARN, Université de Strasbourg, CNRS, IBMC, 67084 Strasbourg, France; b Institute for Biochemistry, Leipzig University, Brüderstr. 34, 04103 Leipzig, Germany

**Keywords:** CCA-adding enzyme, tRNA, Cold adaptation, Psychrophilic protein, X-ray crystallography, SAXS, Psychrophilic RNA polymerase

## Abstract

CCA-adding enzymes are highly specific RNA polymerases that add and maintain the sequence C-C-A at tRNA 3‘-ends. Recently, we could reveal that cold adaptation of such a polymerase is not only achieved at the expense of enzyme stability, but also at the cost of polymerization fidelity. Enzymes from psychrophilic organisms usually show an increased structural flexibility to enable catalysis at low temperatures. Here, polymerases face a dilemma, as there is a discrepancy between the need for a tightly controlled flexibility during polymerization and an increased flexibility as strategy for cold adaptation. Based on structural and biochemical analyses, we contribute to clarify the cold adaptation strategy of the psychrophilic CCA-adding enzyme from *Planococcus halocryophilus*, a gram-positive bacterium thriving in the arctic permafrost at low temperatures down to −15 °C. A comparison with the closely related enzyme from the thermophilic bacterium *Geobacillus stearothermophilus* reveals several features of cold adaptation - a significantly reduced amount of alpha-helical elements in the C-terminal tRNA-binding region and a structural adaptation in one of the highly conserved catalytic core motifs located in the N-terminal catalytic core of the enzyme.

## Highlights

• A high-resolution structure of a psychrophilic RNA polymerase contributes to our knowledge of cold adaptation.
• While catalytic core motifs are conserved, at least one shows cold adaptation.
• Loss of helix-capping increases structural flexibility in a catalytic core motif.
• Overall reduction of alpha-helical elements appears as a strategy for cold adaptation.

## Graphical abstract

**Figure:**
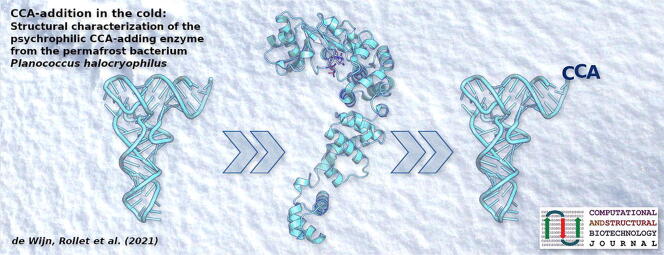

## 1 Introduction

Psychrophilic bacteria are defined as cold-adapted microorganisms thriving at temperatures below 15 °C and tolerating 0 °C [1]. To be functional at temperatures at the freeze point or below, enzymes of such psychrophiles require specific evolutionary adaptations [2]. A widespread strategy is the destabilization of the enzyme/substrate complex to reduce the required activation energy [3], [4], [5]. However, such a destabilization is based on a higher structural flexibility of the enzyme, resulting in an increased temperature sensitivity of cold-adapted enzymes [4]. In general, enzymes do not rely on a uniform strategy for cold adaptation. Rather, structural flexibility can be achieved by a number of factors ranging from a decreased content of secondary structure elements to extended loop regions. In addition, the amount of disulfide bridges, electrostatic interactions and hydrogen bonds can be reduced, while amino acids promoting flexibility (glycine) or interfering with helix formation (proline) are enriched [3], [4], [6], [7], [8], [9], [10].

In DNA and RNA polymerases, a defined conformational flexibility is of vital importance for proper function and correct nucleotide addition [11], [12], [13], [14]. Accordingly, an increased flexibility as a strategy for cold adaptation might interfere with polymerization speed and fidelity. A highly specialized RNA polymerase with an outstanding accuracy is the CCA–adding enzyme (tRNA nucleotidyltransferase) that adds and maintains the triplet sequence C-C-A at the 3′–end of tRNAs [15], [16], [17]. As members of the polymerase β group, CCA-adding enzymes are classified into class I (archaeal enzymes) and class II (bacterial and eukaryotic enzymes) [15], [18], [19]. During synthesis of the CCA sequence, the latter group exhibits defined structural rearrangements in the catalytic core that are required for correct NTP recognition and restricted addition of three positions [12], [14]. Both CTP and ATP are bound in a single nucleotide binding pocket, where highly conserved amino acid residues form Watson-Crick-like hydrogen bonds to the base moiety of the incoming NTP [20]. To switch from CTP towards ATP incorporation, a defined reorientation of these residues is required in order to accommodate the ATP in a highly selective way. As these coordinated movements of the enzyme are essential for a correct and efficient CCA-addition, the increased enzyme flexibility required for activity at low temperatures leads to a conflict between cold adaptation and proper polymerization. Recently, we could show that the CCA-adding enzyme from *Planococcus halocryophilus* (*Pha*CCA), a bacterium from the arctic permafrost growing at –15 °C [21], is such a cold-adapted enzyme and catalyzes CCA-addition *in vitro* down to 0 °C [22]. Interestingly, both the N–terminal catalytic core as well as the C-terminal tRNA–binding region are adapted to the cold, indicating that the psychrophilic features are not restricted to a single protein domain, but are distributed over the whole enzyme. In addition, and consistent with the features of many cold-adapted enzymes, *Pha*CCA shows a decreased thermal stability. However, the price to be paid for this cold adaptation is not only a structural destabilization, but also a significant reduction in polymerization fidelity [22]. Here, we present biochemical and structural data contributing how the psychrophilic CCA–adding enzyme from *P. halocryophilus* is adapted to such low temperatures. Our results reveal that a combination of individual strategies – a global flexibility in the tRNA-binding C-terminus as well as a thermal adaptation in the highly conserved catalytic core motif C controlling interdomain movements during CCA-addition – contribute to the remarkable cold adaptation of this unusual RNA polymerase.

## 2 Materials and methods

### 2.1 Expression of recombinant enzymes

For biochemical analysis, the coding regions of interest were cloned into pET30Ek/Lic and recombinant enzymes were expressed as previously described with N-terminal 6xHis–tag in *E. coli* BL21(DE3) *cca::cam*, lacking the endogenous CCA-adding enzyme in order to avoid any contaminating background CCA-adding activity [22]. Point mutations were introduced into the coding sequence of *P. halocryophilus* CCA-adding enzyme by site-directed mutagenesis using mutagenic primer pairs. Generation of the loop chimera was achieved via amplification of the respective loop coding region from the *G. stearothermophilus* CCA–adding enzyme gene to obtain a megaprimer for QuikChange site-directed mutagenesis in the *Pha*CCA open reading frame [12], [22].

The pETDuet constructs provided both T7 promoter-based expression of *E. coli* RNase T as well as an autonomous expression platform including the native *cca* promotor for comparable expression of the recombinant tRNA nucleotidyltransferase and were established for *in vivo* complementation in *E. coli* strain JM109(DE3) *cca::cam* as described [23]. Transformants were streaked in sectors onto LB agar plates supplemented with ampicillin (100 µg ml^−1^) and were incubated for 2 days at the indicated temperatures.

### 2.2 Preparation and biochemical analysis of *P. halocryophilus* CCA-adding enzyme

Purification of the recombinant enzyme was done according to Ernst *et al*. [22] and stored at 4 °C in 50 mM TRIS/HCl, pH 7.5, 200 mM NaCl, 5 mM MgCl_2_. Protein concentrations were determined using a NanoDrop ND–1000 spectrophotometer with extinction coefficients deduced from their amino acid composition (https://web.expasy.org/protparam/). Samples were ultracentrifuged for 1 h at 100,000 g prior to any further analysis or experiment, and their homogeneity at 20 °C was systematically verified by dynamic light scattering (DLS) using a Zetasizer Nano ZS instrument (Malvern) and a 20 µl quartz cuvette. The reported particle diameter corresponds to the Z–average for at least 5 measurements while fitting a unimodal distribution, as implemented in the instrument. *In vitro* CCA incorporation and CD spectroscopy were performed as described [22].

### 2.3 Electrophoretic mobility shift assay (EMSA)

Electrophoretic mobility shift assays were performed as recently described with minor deviations [24], [25]. 0.3 pmol α–^32^P–ATP-labeled yeast tRNA^Phe^ lacking the CCA-terminus was heated for 2 min at 90 °C, cooled down to room temperature and incubated with 0 to 5 µM enzyme in 50 mM glycine/NaOH (pH 8.5), 10 mM MgCl_2_ and 2 mM DTT for 10 min at temperatures where the enzymes show significant activity (4 °C and 30 °C for *Pha*CCA and 37 °C for *Gst*CCA) [22]. After addition of 80% m/v glycerol to a final concentration of 18.5% m/v, tRNAs were separated on a 5% m/v native polyacrylamide gel for 60 min at 3 W and 4 °C. To visualize complexed and free tRNA substrates, a Typhoon 9410 phosphorimager was used (Cytiva, Freiburg, Germany). Determination of the approximate dissociation constant of *Gst*CCA was performed by nonlinear regression using GraphPad Prism 7.

### 2.4 Crystallization of *Pha*CCA

*Pha*CCA crystals were obtained as described [26]. First diffracting crystals were grown by counter diffusion (CD) in capillaries plunged in 3 M ammonium sulfate, 0.1 M sodium acetate, pH 5.0, from a commercial CD kit (Triana Science & Technologies). Their diffraction patterns extended to a maximum of 2.65 Å and presented an ice-ring at about 3.7 Å. To improve data quality, another condition (1 M diammonium hydrogen phosphate, 100 mM sodium acetate pH 4.5) was used in combination with microseeding [26].

The seed stock was prepared as follows: three droplets of 1 µl containing large number of small crystals (<50 µm) were pooled, resuspended and crushed in 10 µl of the former solution with a standard P10 pipette. Large crystals (150–300 µm) were grown at 20 °C in 24–well plates (NeXtal Biotechnologies) within 1–3 weeks in 1–2 µl droplets made of 1/10th of seed stock, 4/10th of *Pha*CCA and 5/10th of reservoir solutions (in volume ratio).

Prior to data collection, crystals were soaked for 30 s in the reservoir solution supplemented with 20% m/v glycerol, mounted in MicroLoops (MiTeGen) and cryocooled in liquid nitrogen, except crystals grown by CD in ammonium sulfate which were directly cryocooled in their capillary. Crystals of enzyme:ligand complexes were obtained by a preliminary 30 s soaking in the mother liquor containing either 5 mM of CTP or 10 mM of CMPcPP (non-hydrolyzable CTP analog; Jena Bioscience, catalog number NU-438S).

### 2.5 Crystallographic analysis of *Pha*CCA

Crystal characterization was performed at X06DA beamline, Swiss Light Source (Villigen, Switzerland), equipped with a PILATUS 2 M detector or at Proxima 1 beamline, Soleil synchrotron (Saint-Aubin, France), equipped with an Eiger 16 M detector. All data sets were processed and scaled with the XDS Package (Kabsch, 2010), see statistics in Table 1. A selection of 5% of reflections was put apart and used throughout refinement to calculate the R–free value.

**Table 1:** Data collection and refinement statistics.

	CCA-adding enzyme SSAD	CCA-adding enzyme	CCA-adding enzyme + CTP	CCA-adding enzyme + CMPcPP	CCA-adding enzyme + AS
X-ray beamline	SLS - PXIII	PROXIMA1	PROXIMA1	PROXIMA1	SLS - PXIII
Wavelength (Å)	2.075	0.9786	0.9786	0.9786	1.000
Temperature (K)	100	100	100	100	100
Detector	Pilatus 2 M−F	Pilatus 6 M	Pilatus 6 M	Eiger 16 M	Pilatus 2 M−F
Space group	P4(3)2(1)2	P4(3)2(1)2	P4(3)2(1)2	P4(3)2(1)2	P4(3)2(1)2
*a*, *c* (Å)	69.79, 291.21	70.03, 291.59	70.38, 291.49	69.711, 290.342	70.0, 292.2
Solvent content (%)	66.6	66.8	67.2	67.2	66.9
Mean mosaicity (°)	0.08	0.04	0.05	0.11	0.18
Resolution range (Å)	50 – 2.25 (2.31 – 2.25)	49 – 1.80 (1.91 – 1.80)	50 – 1.85 (1.96 – 1.85)	50 – 2.2 (2.34 – 2.2)	50 – 2.80 (2.9 – 2.80)
Total No. of reflections	5,020,925 (116071)	890,444 (139573)	776857(123819)	965,891 (160098)	1,036,294 (104514)
No. of unique reflections	64,470 (4607)	68,555 (10787)	63965(10146)	37,600 (5903)	18,615 (1819)
Completeness (%)	99.0 (96.2)	99.9 (99.3)	99.8 (99.2)	99.8 (99.3)	98.6 (100.0)
Redundancy	78 (25)	13 (13)	12 (12)	25.7 (27.1)	55.7 (57.5)
〈*I*/*σ*(*I*)〉	30.7 (0.9)	19.5 (0.7)	22.1 (1.0)	16.84 (1.98)	21.3 (3.1)
*R*meas (%)	17.0 (311.3)	7.4 (264.8)	6.9 (239.4)	15 (161.6)	25.8 (204)
CC1/2 (%)	99.9 (42.5)	100 (59.0)	100 (45.1)	99.9 (78.6)	99.9 (100.0)
*B* factor from Wilson plot (Å 2)	47.1	44.8	45.0	50.9	59.4
Reflections in working/test sets	34,968/1686	68,408/3420	63,868/3195	35,845/1886	17,701/901
Final *R_work_*/*R*_free_ (%)	18.9/21.1	20.7/23.2	20.2/21.9	20.6/24.2	22.0/25.6
No. of non-H atoms	3251	3310	3398	3214	3030
Protein/solvent/ligand/ion	3062/147/42/0	3043/238/29	3072/268/58/0	3005/99/145/0	2089/21/20/0
R.m.s.d. bonds (Å)/angles (°)	0.008/0.96	0.007/0.794	0.008/0.979	0.011/1.11	0.009/1.05
Average *B* factors (Å^2^)	48.2	44.0	44.0	51.9	53.3
biomolecule/solvent (Å^2^)	48.0/49.9	/42.5	/45.9	51.3/52.7	53.2/46.9
Ramachandran plot regions: most favored, allowed (%)	98.4/1.4	99.0/1.0	99.0/1.0	98.4/1.4	98.1/1.7
PDB id	7OTR	6QY6	6QXN	7OQX	7OTL

The anomalous signal of sulfur atoms present in the native protein (which contains 11 methionine residues, no cysteine) was exploited to determine a set of initial phases using Autosol from PHENIX package [27], [28]. A complete model was built and refined with Autobuild and Phenix_refine.

Other structures were obtained by molecular replacement using the sulfur-SAD model in Phaser [28] and Coot [29] for manual inspection and rebuilding. They were all refined using isotropic Atomic Displacement Parameters (ADPs) and TLS (translation/libration/screw) constraints. Water molecules were added where appropriate, as well as small molecules present in the mother liquor (glycerol, TRIS, acetate, phosphate, sulfate ions). While CTP appeared in a single conformation, two conformations were necessary to model the electron density present in the active site of the enzyme in the case of CMPcPP.

In all models, the residue Val215 was systematically slightly off the Ramachandran and flagged as an outlier due to a local loop conformation. Also, no density could be observed for the sidechain of His35 in the neighborhood of the CTP binding pocket, as well as for the last four residues (374–377) and residues from the N–terminal expression tag (43 residues including the 6xHis-tag). Model building started between position −4 and −2 (corresponding to aspartic acid residues) depending on the quality of the electron density map. Structure analysis and molecular representations were performed with PyMOL Molecular Graphics System, Version 2.4 (Schrödinger, LLC). Structure-based sequence alignments and 2D structure analysis were performed using ENDscript webservice [30].

### 2.6 Small-angle X-ray scattering (SAXS)

SAXS experiments were performed on the SWING beamline at synchrotron SOLEIL (Saint-Aubin, France). The beam wavelength was λ = 1.033 Å. The EigerX4M detector (Dectris) was positioned at a distance of 2,000 mm from the sample with the direct beam off-centered. The resulting exploitable q-range was 0.005 – 0.5 Å^−1^, where the wave vector q = 4π sin θ/λ and 2θ is the scattering angle.

A *Pha*CCA solution (60 µl at a concentration of 2.9 mg/ml) was loaded onto a size exclusion column (Bio SEC-3 with 300 Å pore size, Agilent Technologies) and SAXS measurements were performed throughout elution operated at 15 °C and a flow rate of 0.3 ml/min with a mobile phase containing 100 mM HEPES/NaOH, pH 7.5, 150 mM NaCl and 10 mM MgCl_2_. The eluate was analyzed by SAXS in a continuous flow capillary cell with a frame duration of 990 ms at intervals of 10 ms. Data processing, interpretation, Rg evaluation over elution profiles were performed using Foxtrot [31] and data analysis (determination of Rg, Dmax) with the ATSAS package [32]. A complete model including the N–terminal extension was generated using Modeller [33] based on the crystal structure. It was refined under SAXS constraints using DADIMODO [34], a genetic algorithm-based refinement program, to better fit the SAXS profile owing to a goodness–of–fit calculated with CRYSOL [35]. Graphics were prepared with LibreOffice Draw (The Document Foundation) and BioXTAS-RAW [36].

### 2.7 Nanoscale differential scanning fluorimetry (nanoDSF)

Comparative thermal stability assays were carried out by nanoDSF in a Prometheus NT.48 instrument (NanoTemper Technologies). CCA-adding enzymes were incubated on ice at a concentration of 10 nM in their storage buffer, alone or in the presence of 30 nM of either ATP, CTP or GTP 15 min prior to experiment. Glass capillaries were filled with 10 μl of each solution and deposited on the sample holder. Excitation wavelength was set to 280 nm and intrinsic tryptophan fluorescence was recorded at 330 and 350 nm at temperatures ranging from 20 to 95 °C (1 °C/min, 1 measurement per 0.044 °C). Corresponding first derivative plots were calculated using the manufacturer’s software (PR.ThermControl, version 2.1.2) to determine melting temperature (Tm), along with those of the scattering signal highlighting the unfolding of the proteins.

## 3 Results

### 3.1 Structural characterization of *Pha*CCA

**Fig. 1:**
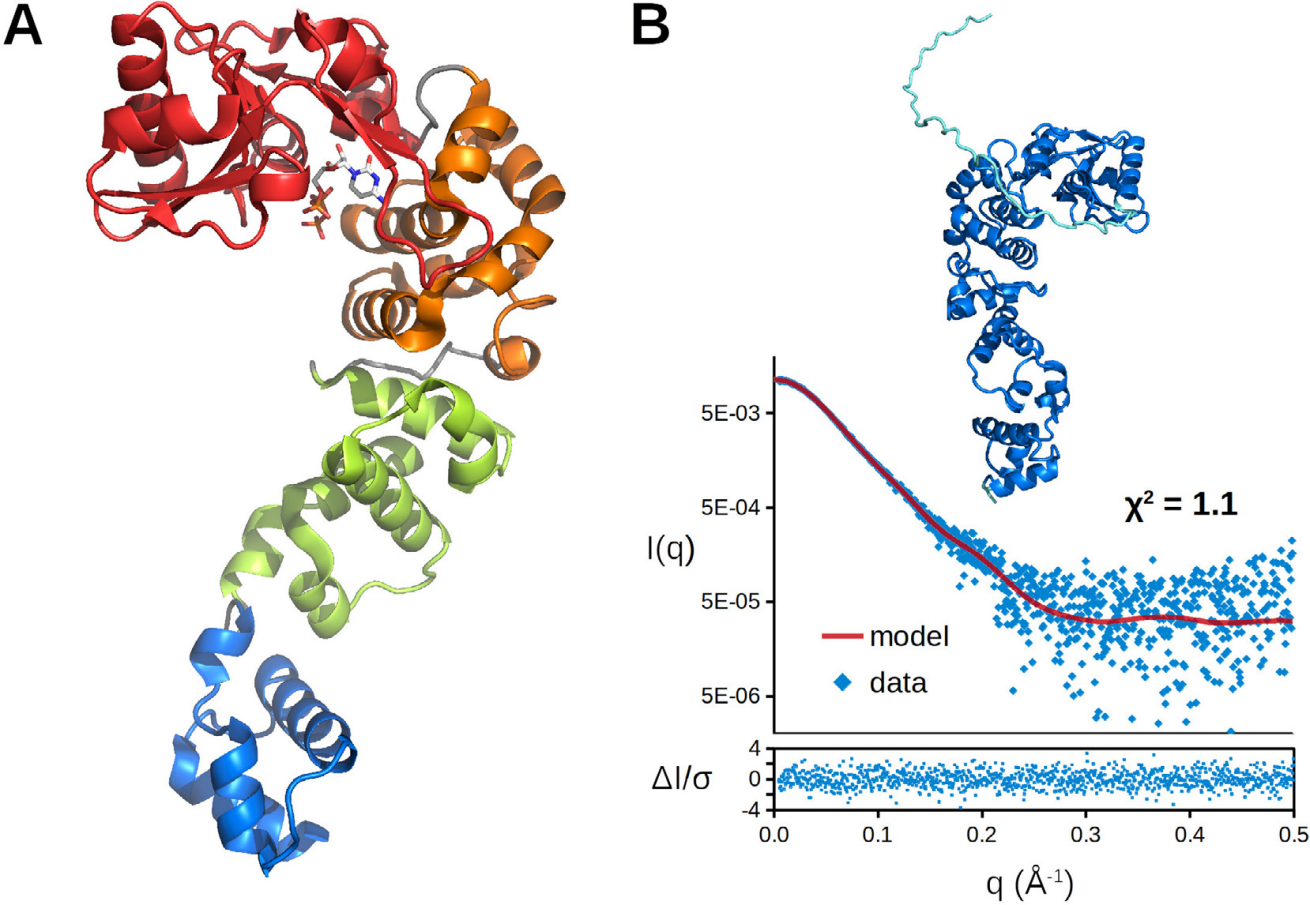
Structural organization of psychrophilic *Pha*CCA. A) Crystal structure of *Pha*CCA in complex with CTP determined at 1.85 Å resolution. The enzyme monomer presents the characteristic seahorse shape similar to that described by Li et al. [20], with its head, neck, body and tail domains depicted in red, orange, green and blue, respectively. B) Solution structure of *Pha*CCA including the enzyme core (dark blue) and the flexible 43 residues long N–terminal expression tag (cyan). The theoretical SAXS profile (red line) corresponding to this monomeric model is in good agreement with the experimental data (blue dots), as indicated by the goodness-of-fit (χ^2^). The error-weighted residual difference plot *ΔI/σ = [I_exp_(q) – I_model_(q)]/σ(q)* is given at the bottom. (For interpretation of the references to colour in this figure legend, the reader is referred to the web version of this article.)

In order to investigate the structural properties of *Pha*CCA leading to cold adaptation, crystals of the corresponding recombinant enzyme were grown. The crystallized construct consisted of the *P. halocryophilus* wild type sequence (377 amino acids) with an N–terminal extension of 43 residues including a 6xHis affinity tag. First crystals of *Pha*CCA were obtained in the presence of ammonium sulfate as crystallant and diffracted to a maximum resolution of 2.8 Å [26]. They were used as seeds to screen for new conditions and led to crystals with similar cell parameters and symmetry (see Table 1) but showing significantly improved diffraction properties (extending to a resolution of 1.8 Å) in the presence of PEG 3350 or diammonium hydrogen phosphate. The latter crystallant was selected in combination with microseeding to ensure a reproducible crystal production [26]. Sulfur-SAD data collection was performed to determine a set of phases and calculate an experimental electron density map, which allowed to automatically build > 90% of *Pha*CCA residues (Fig. S1A–B) and revealed the characteristic architecture of bacterial and eukaryotic CCA-adding enzymes (Fig. 1) and its seahorse shape popularized by Li *et al*. [20].

An ensemble of five structures was determined under cryoconditions, in apo state or in complex with a substrate in the presence of various salts (Fig. S1C–E). All crystals showed a high solvent content (67%) with one enzyme monomer in the asymmetric unit. The refined structures are very similar, showing a root mean-square deviation (rmsd) ranging from 0.16 to 0.22 Å (for 370 superimposed Cα positions) when compared to the reference apo structure solved at the highest resolution (Fig. S2). Their N–terminal extension is disordered and the first visible residue varied from position −4 to −2 at the end of the extension. The four C-terminal residues (HSHT) are also disordered, as well as the central part of the flexible loop (residues 82–93) in the head domain, except for the complex with CTP where it is clearly visible (Fig. S1C).

Fast crystal soaking (30 s) with CTP or its non-hydrolyzable analog prior to cryocooling readily led to the binding of the ligand in the enzyme’s catalytic site (Fig. S3). In contrast, soaking or co–crystallization attempts with ATP were not successful.

Early crystal structures of CCA-adding enzymes from *G. stearothermophilus* and *H. sapiens* were reported as dimers [20], [37], but dimer interfaces were differing, and the existence of a functional dimeric enzyme has never been confirmed. *Pha*CCA is unambiguously monomeric in its crystalline state as well as in solution, as shown by SEC and SAXS analyses (Fig. 1B). Gyration radius *Rg* derived for *Pha*CCA from the Guinier plot is 30.7 Å (Fig. S4), in agreement with the theoretical value calculated for the crystal structure of the monomer (28.2 Å) to which a flexible N-terminal extension is appended. Also, the Kratky plot is consistent with a molecule showing compact multidomain core and a flexible tail (Fig. S4).

### 3.2 Psychrophilic adaptation of *Pha*CCA

**Fig. 2:**
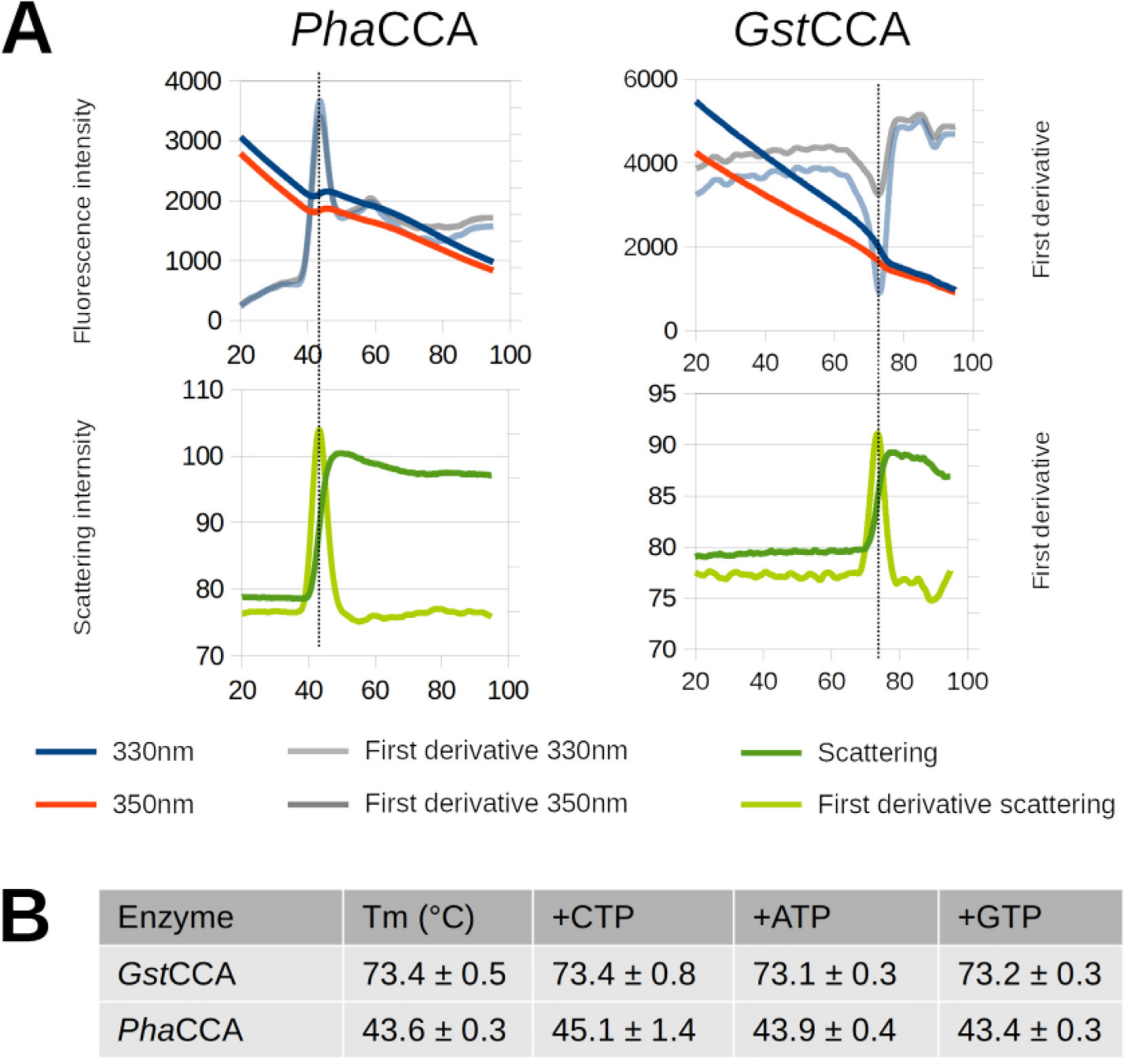
Comparison of *Pha*CCA and *Gst*CCA thermal stability by NanoDSF. A) Representative curves are shown for the two apo enzymes with the variation of fluorescence at 330 and 360 nm, as well as the scattering signal, whose increase confirms protein unfolding. Melting temperatures (Tm) correspond to the inflection point of the first derivatives. B) Tm values in the absence and presence of nucleotides, and associated standard deviations calculated as the mean of three values (Tm from 330 nm, 350 nm fluorescence and from scattering) for at least 3 independent samples.

*Pha*CCA was compared to a close homolog from *Geobacillus stearothermophilus* (*Gst*CCA), a bacterium belonging to the order of *Bacillales* like *P. halocryophilus* [20]. The obtained thermal stability profiles determined by NanoDSF are typical for psychrophilic and thermophilic enzymes with Tms of ∼ 43 °C *vs* 73 °C, respectively (Fig. 2). These values are consistent with Tm values obtained by CD ([22] and below). The slight Tm variation observed between the two methods is likely due to the fact that they track different steps of unfolding (the loss of 2D structures in CD, the increase of solvent exposure and resulting fluorescence quenching of aromatic residues in NanoDSF) and was already described for other enzymes [38]. Because small ligands can potentially stabilize a protein fold upon binding and induce an increase in Tm, the analysis was reproduced in the presence of nucleotides. However, no significant effect was observed with the natural substrates CTP and ATP or with GTP, which is not supposed to bind. Still, it can be an indication that CTP and ATP binding does not induce conformational changes large enough to alter the enzyme’s stability (see Fig. S4).

**Fig. 3:**
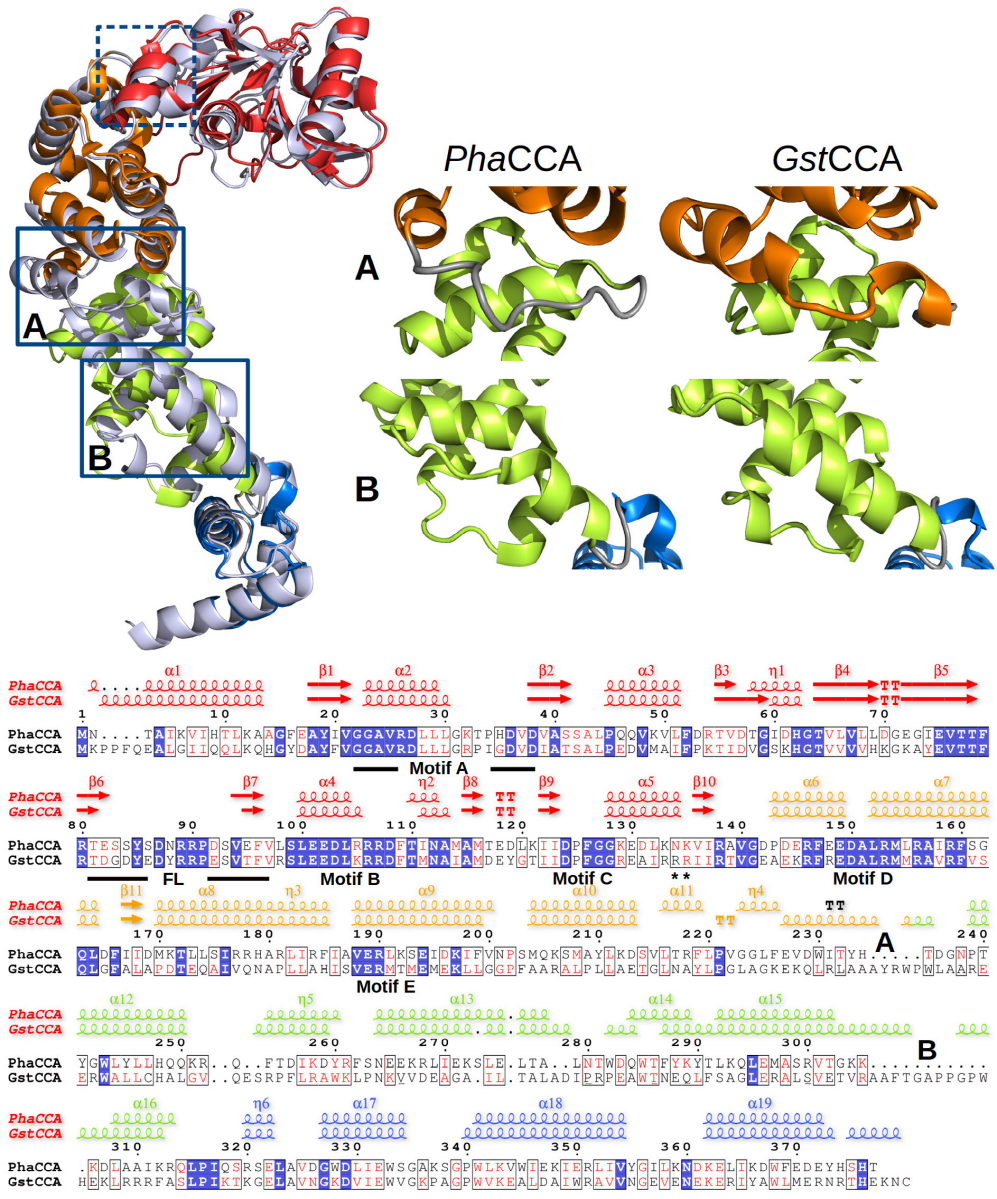
Structural adaptations in the psychrophilic CCA-adding enzyme. On the left, the structural alignment of the *Pha*CCA enzyme (with its domains colored as in Fig. 1) and its thermophilic homolog *Gst*CCA [20] depicted in light blue, reveals typical cold adaptations in its structure. Examples of shorten alpha-helical elements in *Pha*CCA are boxed in blue (the dashed box highlights helix α5 in motif C discussed in Fig. 5). As illustrated by the close-up views on the right, the helix α11 in region A at the junction of the neck and body is replaced by a loop, while in region B in the body domain helices α15-16 are drastically reduced. These variations in helical regions can be easily spotted in the 2D structure elements presented above the sequence alignment for each structure (see labels A, B). Strictly conserved residues are highlighted in blue, similar residues in red. Catalytic core motifs A to E and the flexible loop (FL) are indicated. Asterisks indicate helix-capping residues R137 and R138 of *Gst*CCA. (For interpretation of the references to colour in this figure legend, the reader is referred to the web version of this article.)

The structures of *Pha*CCA and *Gst*CCA were compared to track signs of structural adaptation to cold. As shown in Fig. 3, they can be easily superimposed with an RMSD of 2.3 Å (taking into account 360 Cα positions). The sequence alignment in Fig. 3 illustrates the high sequence similarity in the N–terminal region, where the catalytic core is located in the head and neck domains, displaying the characteristic motifs A to E, as well as a flexible loop between motifs A and B. In this region, the two structures show comparable 2D structure contents with 58.8% and 59.0% of residues located in alpha-helical elements (Table 2). In contrast, the C-terminal region, encompassing the body and the tail domains and constituting the tRNA binding site, is much less conserved and its helix content (alpha and 3_10_ helices) shows a clear 14% drop in *Pha*CCA (71.7%) compared to *Gst*CCA (86.1%) (Table 2). An example of drastic helix reduction is illustrated in Fig. 3, with complete helices replaced by a loop in *Pha*CCA at the junction of the neck and the body domains, as well as in the body. To investigate whether this alpha-helical reduction affects the affinity of *Pha*CCA to a tRNA substrate, electrophoretic mobility shifts were performed (Fig. S5). For *Gst*CCA, an interaction with a tRNA substrate was clearly visible, resulting in a Kd value of approximately 1.3 µM, comparable to that of other class II CCA-adding enzymes [24], [25]. In contrast, no gel shift was visible for *Pha*CCA, indicating a reduced tRNA interaction that does not allow a calculation of a binding constant. Taken together, while the reduced number of alpha-helices in the tRNA-binding C-terminus seems to decrease the substrate affinity of *Pha*CCA, it is likely to represent - in combination with elongated loop elements - a main feature of cold adaptation that provides the increased structural flexibility observed in this enzyme [22].

**Table 2:** 2D structure content of psychrophilic and thermophilic enzymes.

Enzyme	*Pha*CCA	*Gst*CCA
PDB id	6QY6	1MIW
Total number of residues	377	404
Number of residues in helices (α and 3_10_)	241	280
Number of residues in β-strands	37	39
Number of residues in 2D structures	278	319
**% of residues in helices overall**	**63.9**	**69.3**
% of residues in β-strands overall	9.8	9.7
% of residues in 2D structures overall	73.7	79.0
% of residues in helices in head + neck	58.8	59.0
% of residues in helices in body + tail	**71.7**	**86.1**

In contrast, the N–terminal catalytic core seems to retain all sequence and structure characteristics of bacterial CCA–adding enzymes. Yet, our previous investigations clearly indicated that this enzyme region also contributes to the psychrophilic adaptation of *Pha*CCA [22]. As structural flexibility is a prominent prerequisite for cold adaptation, we focused on regions in the N–terminal catalytic core that participate or contribute to structural rearrangements during CCA–addition, in particular the nucleotide binding pocket [20], the flexible loop [39] and the hinge element of motif C [12].

**Fig. 4:**
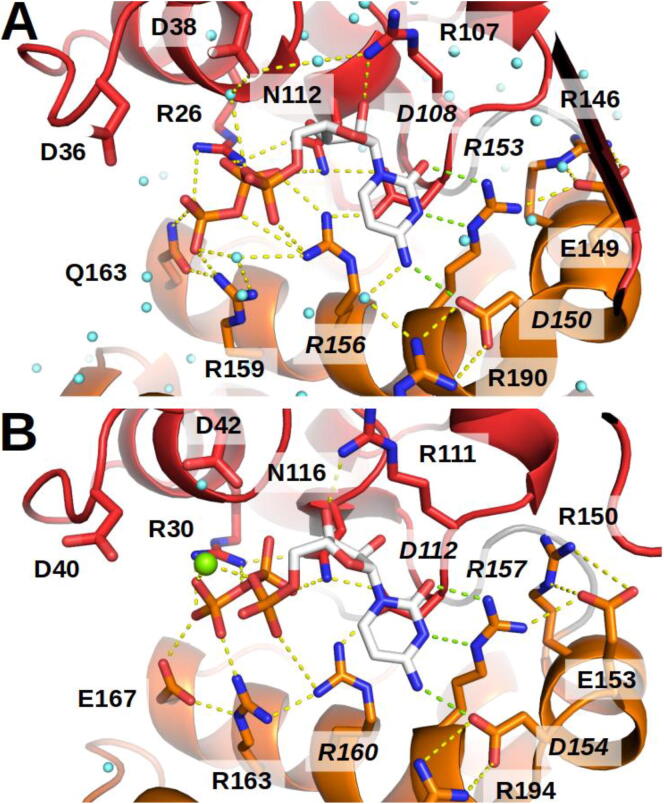
A highly conserved CTP binding pocket. Close-up views of the catalytic core of *Pha*CCA (A) and *Gst*CCA (B) in the presence of CTP. Highly conserved residues with side chains contacting the nucleotide are indicated. Water molecules are represented as blue spheres and magnesium ion bound to *Gst*CCA as a green sphere. No magnesium site could be identified in *Pha*CCA structure. The dense network of interactions (with a distance < 4 Å) between protein side chains and the substrate is symbolized by dashed lines. Four side chains indicated in italics interact directly with the nucleobase and constitute the amino acid template in motif D: two couples of Arg/Asp residues, one forming a stacking platform below the base, the second making three hydrogen bonds indicated by green dashed lines with the Watson-Crick face of CTP. The latter residues are hold in place by additional connections to surrounding side chains. In both structures, the orientation of the CTP is almost identical and the amino acid template of motif D forms the same interactions to the bound base moiety, indicating that the templating function is not specifically cold adapted in *Pha*CCA. (For interpretation of the references to colour in this figure legend, the reader is referred to the web version of this article.)

When the *Pha*CCA crystals were soaked with CTP or a non-hydrolyzable CTP analog, the diffraction patterns of the resulting co–crystals revealed an orientation of the bound nucleotide that is identical to that found in the corresponding *Gst*CCA/CTP structure [20] (Fig. 4 and S4). Hence, the shape of the nucleotide binding pocket with the base-recognizing residues EDxxR of motif D does not differ between the thermophilic and the psychrophilic enzymes, excluding a prominent role in temperature adaptation.

A comparison of the apo enzyme with the two complexes shows that CTP binding does not involve any conformational change and the substrate is almost accommodated in a key-and-lock manner (Fig. S4). This is in agreement with the very low RMSDs between the different structures (Fig. S2).

As indicated above, our attempts to obtain *Pha*CCA crystals with ATP remained unsuccessful, and the ATP-bound state that could have been compared to the *Gst*CCA:ATP complex was not observed.

**Fig. 5:**
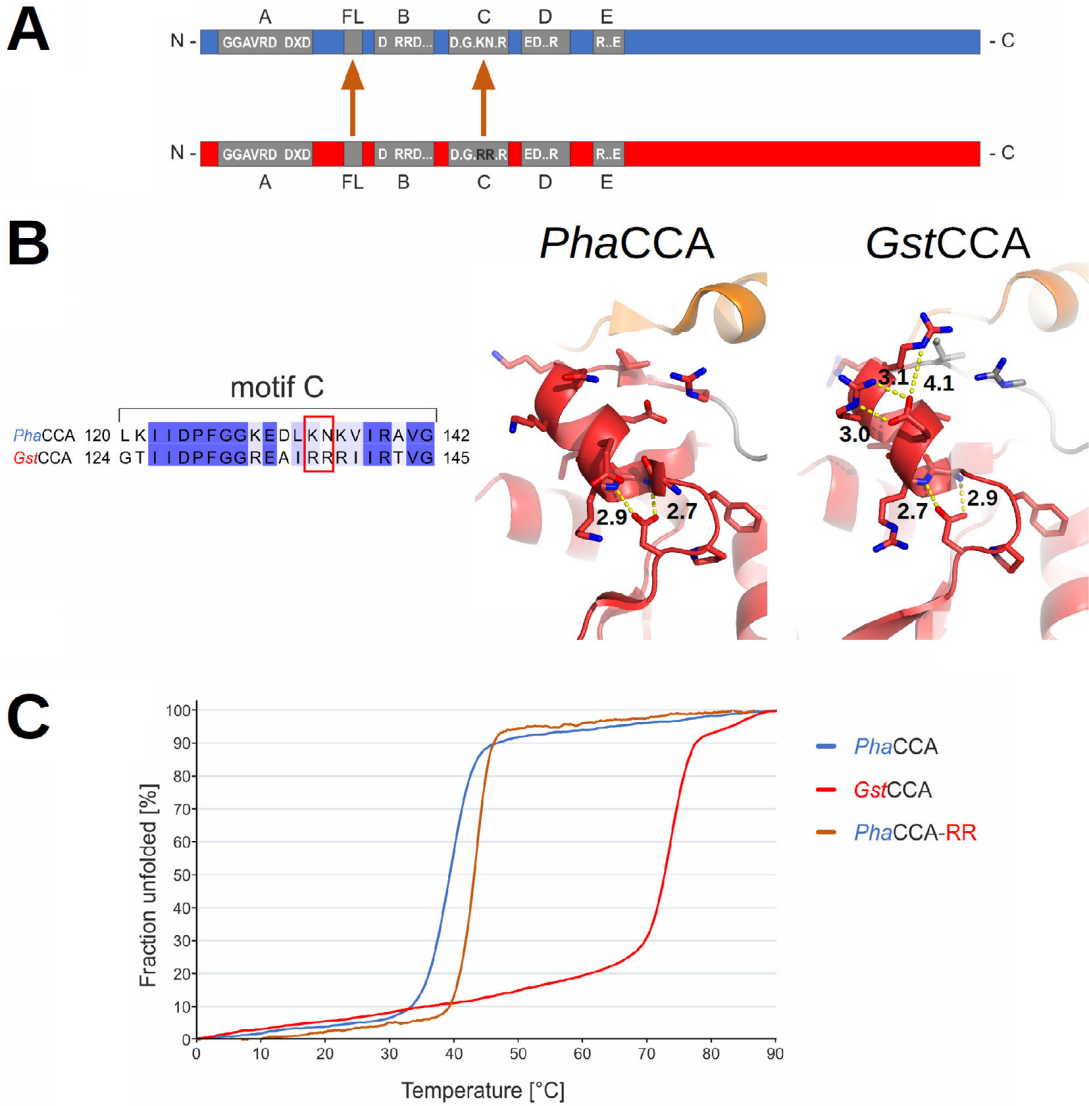
Replacement of flexible elements in *Pha*CCA by their thermophilic counterparts. A) Bar diagrams of *Pha*CCA (blue) and *Gst*CCA (red). Elements of the catalytic core are indicated in grey. In *Pha*CCA, the flexible loop (FL) and motif C were replaced by the corresponding regions of *Gst*CCA. B) In *Gst*CCA, helix α5 in motif C (see Fig. 3) carries two R residues that are absent in *Pha*CCA (left panel). This arginine pair stabilizes an alpha-helical element by a C-terminal helix-capping (right panel). Note the presence of a conserved N-terminal helix-capping involving a strictly conserved aspartic acid of motif C. C) Thermal unfolding of CCA-adding enzymes and *Pha*CCA with introduced arginine positions involved in C-terminal helix-capping. With a melting temperature of approximately 39 °C, *Pha*CCA is a true psychrophilic protein (blue). Introducing the helix-capping RR pair increases the melting temperature of *Pha*CCA–RR to 43 °C (brown). *Gst*CCA shows a typical thermophilic profile and melts at 73 °C (red). (For interpretation of the references to colour in this figure legend, the reader is referred to the web version of this article.)

The reorganization of the amino acid template in motif D during the specificity switch from CTP to ATP binding is mediated by a flexible loop element [39], [40]. It is discussed that this element, located between motifs A and B, acts as a lever to adjust the base-pairing amino acids for adenine interaction after the addition of two C residues to the tRNA substrate [41], [42]. To investigate a possible contribution of the flexible loop to cold adaptation, we replaced this element in *Pha*CCA by the corresponding region of the thermophilic *Gst*CCA enzyme (Fig. S6A). While the loop sequence itself is not very conserved [41], it is flanked by constant sequence elements, allowing the identification of compatible fusion sites in both enzymes. As the N–terminal border, we selected the position separating the basic and the acidic residue in the conserved B/A motif (*Pha*CCA: T81, *Gst*CCA: T85), while the C-terminal border was the last variant position of the loop region (*Pha*CCA: L98, *Gst*CCA: R102), resulting in the loop chimera *PGP*CCA (PGP: first and last character describe the origin of the enzyme body, the second character indicates the origin of the loop) (Fig. 5B and S5B). CD spectral analysis of the recombinant protein revealed no differences in overall secondary structure compared to the parental enzymes. In addition, *PGP*CCA was fully active *in vitro* and added a complete CCA triplet on a yeast tRNA^Phe^ transcript, a standard substrate for *in vitro* CCA–addition (Fig. S7). These data indicate that the loop replacement did not interfere with proper protein folding and catalytic activity. To determine a possible impact on thermal stability, unfolding of the *PGP*CCA chimera was investigated by CD spectroscopy at 222 nm over a temperature gradient ranging from 0 to 90 °C [22]. With a denaturation curve identical to that of *Pha*CCA, the chimera showed no differences to the parental enzyme (Fig. S6C). These data show that the flexible loop apparently does not contribute to the cold adaptation of the *Pha*CCA enzyme.

The third N-terminal element contributing to flexibility is motif C, a region acting as a spring element involved in the reorientation of head and neck domains during CCA-addition [12]. The sequence alignment exhibits a high degree of similarity between *Pha*CCA and *Gst*CCA (Fig. 3, Fig. 5B). The side chain of a strictly conserved aspartate – D124/D128 in *Pha*CCA/*Gst*CCA, respectively – makes a stabilizing interaction with the backbone of the two N–terminal residues of helix α5. This feature is described as helix-capping and is known to contribute to protein stability [43], [44], [45]. At the opposite extremity of this helix, the two arginine residues (R137, R138) interact with E134 in the crystal structure of *Gst*CCA. This corresponds to another feature of helix capping with side chains of amino acids at positions C1 and C2, stabilizing the alpha–helical structure through interaction with amino acids within the helix [46], [47]. In the thermophilic *Gst*CCA, the C1 and C2 residues form ionic bridges with E134 (Fig. 5B). This feature is absent in *Pha*CCA, where the arginines are replaced by K133 and N134. To investigate a potential cold adaptation of motif C in *Pha*CCA in more detail, K133/N134 in *Pha*CCA were replaced by the corresponding *Gst*CCA positions R137 and R138, resulting in *Pha*CCA–RR (Fig. 5A). Similar to the loop chimera, *Pha*CCA–RR was catalytically active in CCA-addition, and CD spectral analysis indicated that its structure does not deviate from that of the corresponding wild type enzyme (Fig. S7). However, thermal denaturation analysis revealed that the two helix-capping arginines from *Gst*CCA lead to a stabilization of the *Pha*CCA structure, increasing the melting temperature by approximately 4 °C (Fig. 5C). It is interesting to note that the stabilizing effect of single amino acids in motif C has also been observed for the structurally related human CCA–adding enzyme. Here, replacement of aspartate at position 139 by alanine abolished the N–terminal helix capping and led to a strongly reduced A–adding activity [12]. Taken together, whereas the nucleotide binding pocket appears unchanged and the flexible loop is not involved in cold adaptation of *Pha*CCA, the absence of C-terminal helix-capping residues in motif C seems to contribute to psychrophilic adaptation at the price of a simultaneously reduced tRNA substrate binding.

### 3.3 Thermostabilizing effect of *Pha*CCA-RR is not sufficient *in vivo*

**Fig. 6:**
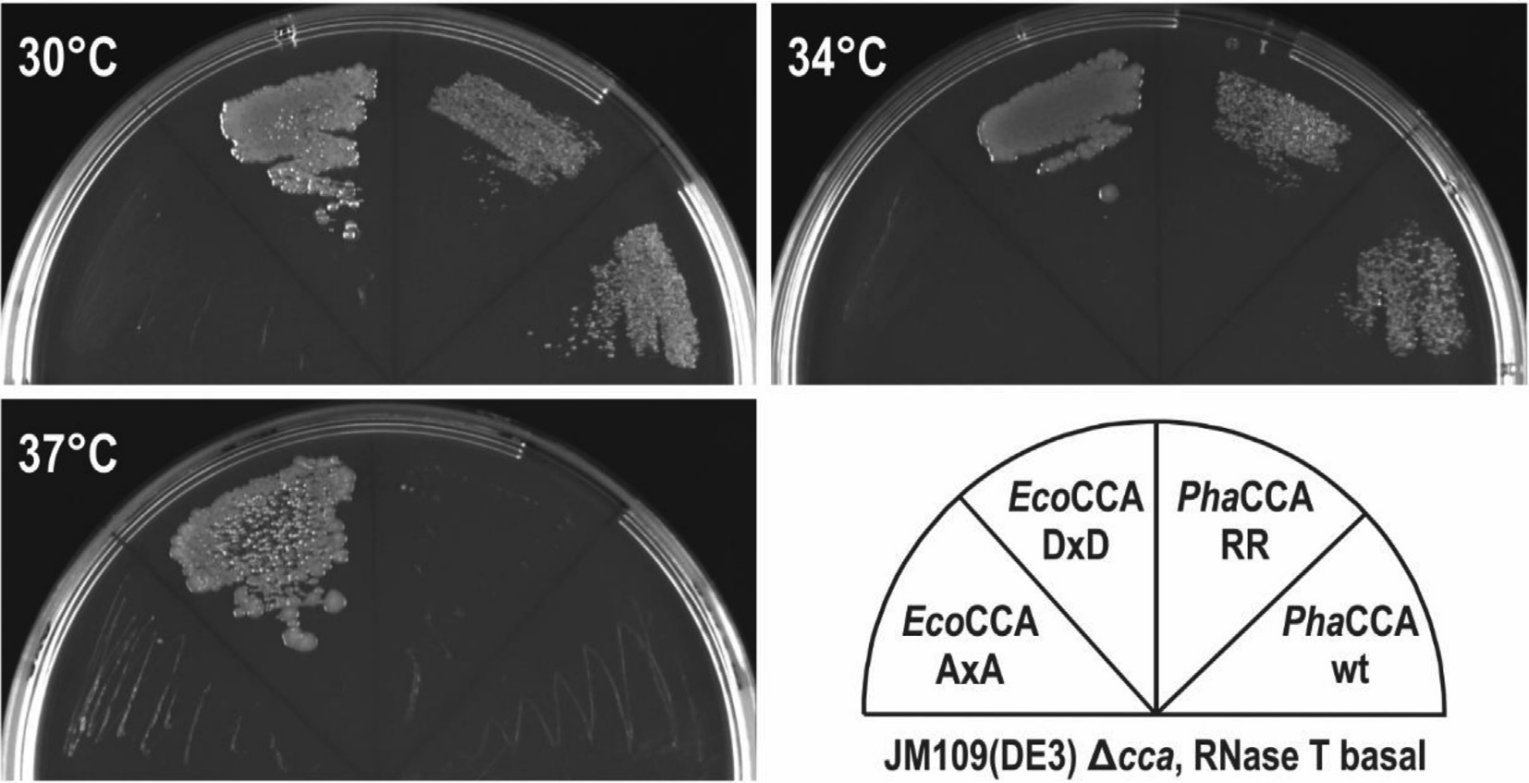
Temperature-dependent activity of *Pha*CCA–RR *in vivo*. *Pha*CCA–RR complements for the endogenous CCA-adding activity in *E. coli* at temperatures up to 34 °C. At 37 °C, however, the enzyme is not sufficiently active, indicating that the helix-capping effect does not provide enough stability to render the enzyme active at higher temperatures. *Eco*CCA, *E. coli* CCA–adding enzyme; DxD, catalytically active wild type enzyme; AxA, inactive *Eco*CCA variant; *Pha*CCA–RR, *P. halocryophilus* CCA–adding enzyme with K133R and N134R replacements; wt, wild type.

To investigate their temperature-dependent catalytic activity in a natural environment, both *Pha*CCA wild type and *Pha*CCA–RR enzyme were subjected to *in vivo* complementation in a *cca*–deficient *E. coli* strain (Fig. 6). To this end, the corresponding genes were cloned into a previously described pETDuet-1-based dual expression system [23]. This system allows to study an enzyme’s ability to repair truncated tRNA CCA–ends. A catalytically inactive version of the *E. coli* enzyme (*Eco*CCA AxA) does not complement and served as negative control when evaluating growth fitness behavior. Introduction of the *Pha*CCA constructs revealed that both *Pha*CCA wt and *Pha*CCA–RR promote growth at 30 °C. To examine whether the increased thermostability of *Pha*CCA–RR is reflected by a CCA–repair activity at elevated temperatures, transformants were incubated at 34 and 37 °C. Interestingly, we observed no differences in growth behavior between wt and RR variant at the investigated temperatures. While both enzyme versions enabled the cells to thrive at 34 °C, no growth was observed at 37 °C. Obviously, the structure-stabilizing effect of the helix-capping R residues is not sufficient for proper activity at this temperature, and it is likely that other catalytically important regions of *Pha*CCA suffer from subtle unfolding that abolish catalytic activity.

## 4 Discussion

CCA-adding enzymes constitute a specific class of RNA polymerases that function without nucleic acid template. They can accommodate all tRNAs present in the cell to ensure that they carry an intact 3′–CCA tail necessary for aminoacylation. Synthesis of this CCA sequence is accompanied by conformational changes in the enzyme to correctly position the tRNA for each polymerization step and select the correct nucleotide [12], [13], [14]. This work provides the first detailed insight into the structure of a psychrophilic member of this polymerase family. Cold-living organisms are known to produce more flexible enzymes in order to lower their activation energy [2] and the central question for CCA-adding enzymes is how this adaptation can be achieved without compromising the specificity of the reaction, which requires an accurate selection of incorporated nucleotides. To address this question, we compared the structural properties of the psychrophilic *Pha*CCA and the thermophilic *Gst*CCA, both originating from bacteria belonging to the order of *Bacillales*. With a Tm difference of 30–34 °C (Fig. 2, Fig. 5), their thermal stability profile is characteristic of their respective ecosystem. Their evolutive proximity (39% sequence identity overall, 50% in the catalytic head and neck domains) allows to easily spot 1D-2D-3D structural differences as illustrated in Fig. 3. While *Pha*CCA shows the characteristic shape of bacterial CCA-adding enzymes, it also presents clear signs of cold adaptation. Over the past two decades, a variety of psychrophilic enzymes has been compared to their mesophilic and thermophilic homologs, showing that the global reduction of the number of non-covalent interactions (H-bonds and salt bridges) is a general hallmark of their adaptation. This is commonly correlated with a loss of 2D structure elements, a reduction of arginine residues involved in ion pairings on the protein surface and of the size of non-polar side chains in hydrophobic clusters as well as clusters of glycines instead of prolines in loops in order to increase the flexibility of enzymes and their activity at low temperatures [2], [7], [8], [9], [48], [49], [50], [51], [52], [53]. Variations in amino acid composition, such as shifts from arginine to lysine or proline to glycine, are not seen in *Pha*CCA, but the ratio of negatively to positively charged residues is slightly increased, causing a drop of pI to 6.1 (8.4 for *Gst*CCA), which is another characteristic of psychrophilic proteins. The most obvious feature is the reduction of helical structures (Fig. 3) at the hinge between N- and C-terminal regions of the enzyme, as well as in the C-terminal part involved in tRNA interaction and positioning. The local weakening of hydrogen bond network resulting from replacing helices by loops certainly provides additional flexibility to the body domain and, considering its central position, to the whole enzyme, which functions as a clamp or vise to position and check the tRNA substrate status [54], [55].

The N-terminal region is much more conserved and secondary structure contents are very similar in *Pha*CCA and *Gst*CCA. Hence, this region confers substrate specificity and is responsible for the catalytic activity. It is interesting to note that CTP binding occurs without structural change (Fig. S4) and the strict conservation of the amino acids forming Watson-Crick-like hydrogen bonds to the incoming nucleotide [20] excludes any cold specific adaptation of the nucleotide binding pocket. However, the specificity switch from CTP to ATP is clearly affected. At 4 °C, the Km value for ATP incorporation is 64-fold higher than that of *Gst*CCA, suggesting a strongly reduced binding affinity for ATP [22], in accordance with the impossibility to soak this nucleotide in the crystalline enzyme. This - as well as the observed reduced tRNA affinity - is also in line with the common adaptation strategy of cold-adapted enzymes: the destabilization of the enzyme/substrate complex leads to a reduction of the required activation energy [3], [4], [5].

In bacterial and eukaryotic CCA-adding enzymes, different flexible regions are important for highly coordinated movements during nucleotide addition as well as to monitor the structural intactness of the tRNA substrate [12], [13], [14], [55]. To switch the enzymes’ specificity from CTP towards ATP, the templating amino acids of the nucleotide binding pocket need to be reoriented, which demands for a rearrangement of the N–terminal region [20], [23], [56], [57], [58]. In this transition, the flexible loop acts as a lever that forces the amino acid template into its base-specific specific position [39], [41]. While similar surface-exposed flexible loops are often targets for the thermal adaptation of proteins [8], our data indicate that the flexible loop of *Pha*CCA does not contribute to its activity in the cold.

In contrast, the second flexible element motif C, which coordinates interdomain rearrangements during catalysis, is an interesting spot of adaptation [12]. It presents a C-terminal helix capping in *Gst*CCA, while this stabilizing interaction is absent in *Pha*CCA. When C1 and C2 residues are restored, *Pha*CCA gains 4 °C of Tm, reinforcing the idea that the identity of these side chains contributes to the cold adaptation of this enzyme. These results are in line with data from other psychrophilic enzymes, where N- or C-caps have been found to weaken alpha-helices in order to adapt to low temperatures [6]. In addition, single amino acid substitutions in the near active site of a psychrophilic adenylate kinase lead to an increase in T_m_ by ∼ 8 °C [12], [52]. In conclusion, our results indicate that the highly conserved motif C is not only essential to ensure proper CCA-addition, but is equally important to modulate the temperature stability in psychro- and thermophilic enzymes.

Although the introduction of two helix-capping amino acids from *Gst*CCA into *Pha*CCA (*Pha*CCA–RR) led to thermostabilization of the *Pha*CCA variant *in vitro*, no such effect could be observed when complementing under natural conditions in *E. coli* (Fig. 6). Possibly, the thermostabilizing effect observed *in vitro* is not sufficient for the rather stringent *in vivo* system demanding for an efficient CCA-repair activity [23]. Further, motif C destabilization is probably not the only cold adaptation in *Pha*CCA, and additional cumulative adaptations distributed over the whole protein render the enzyme truly psychrophilic. As a result, such regions are obviously destabilized at increased temperature, and their unfolding inactivates *Pha*CCA-RR, despite an increased stability of motif C due to the introduced helix-capping arginine residues. This is supported by the fact that chimeric enzymes consisting of N- and C-terminal halves from *Pha*CCA and *Gst*CCA show temperature adaptations in both regions [22]. Such cumulative structural changes involved in adaptation to temperature have also been reported elsewhere, such as in isocitrate dehydrogenase, which - depending on the respective bacterium - has up to three different regions contributing to the thermal properties [59].

Taken together, the crystallographic and biochemical analysis of the psychrophilic *Pha*CCA enzyme shows that cold adaptation is not only achieved by the local uncapping of an alpha-helix in the catalytic core but also by a general reduction of alpha-helical parts in the C-terminus of this enzyme. While it is a point of debate whether proteins are cold-adapted due to an increase in global or local flexibility [60], [61], the CCA-adding enzyme of *P. halocryophilus* seems to make use of both strategies.

## 5 Conclusion

Our structural data contribute to clarify the mechanism of cold adaptation in the tRNA nucleotidyltransferase of *P. halocryophilus*. We confirm recent observations indicating that this enzyme acquired increased flexibility as a strategy for cold adaptation [22]. The crystal structures reveal a globally conserved scaffold with local adaptations to provide the structural plasticity compatible with catalysis at low temperature. The clearly visible reduction of helical regions appears in the less conserved C-terminal part of the enzyme that shows promiscuous binding of all tRNAs as substrates. The N-terminal catalytic core, however, which is catalytically active and shows high substrate specificity for CTP and ATP, is only marginally affected, though lacking a helix-capping feature that potentially brings 4 °C of thermal stability. Further investigations will clarify whether other RNA polymerases follow a similar adaptive strategy for efficient synthesis in the cold.

## 6 Funding information

This work used the Integrated Structural Biology platform of the Strasbourg Instruct-ERIC (PID 12797) center IGBMC-CBI supported by FRISBI (ANR-10-INBS-05–01). This work was supported by the Deutsche Forschungsgemeinschaft (grant no. Mo 634/10–1 to M.M.), the French Centre National de la Recherche Scientifique (CNRS), the University of Strasbourg, the LabEx consortia “NetRNA” (ANR-10-LABX-0036_NETRNA to C.S.), a PhD funding to R.dW. from the Excellence initiative (IdEx) of the University of Strasbourg in the frame of the French National Program “Investissements d’Avenir”, a PhD funding to K.R. from the French-German University (DFH-UFA grant no. CT-30–19). The authors benefitted from the PROCOPE Hubert Curien cooperation program (French Ministry of Foreign Affair and DAAD to C.S. and M.M.).

## Declaration of Competing Interest

The authors declare that they have no known competing financial interests or personal relationships that could have appeared to influence the work reported in this paper.
