# Supplementary material for: CCA-addition in the cold: Structural characterization of the psychrophilic CCA-adding enzyme from the permafrost bacterium Planococcus halocryophilus
Source: Comput Struct Biotechnol J. 2021 Oct 21;19:5845–55. doi: 10.1016/j.csbj.2021.10.018 (PMC8563995; doi:10.1016/j.csbj.2021.10.018)
Supplement: Supplementary data 1 [file mmc1.docx]

**Supplementary material**

**CCA addition in the cold: Structural characterization of the psychrophilic CCA-adding enzyme from the permafrost bacterium *Planococcus halocryophilus***

Raphaël de Wijn^a,#^, Kévin Rollet^a,b,#^, Felix G.M. Ernst^b^, Karolin Wellner^b^, Heike Betat^b^, Mario Mörl^b*^, Claude Sauter^a*^

^a^Architecture et Réactivité de l’ARN, Université de Strasbourg, CNRS, IBMC, 67084 Strasbourg, France

^b^Institute for Biochemistry, Leipzig University, Brüderstr. 34, 04103 Leipzig, Germany

^#^these authors contributed equally to the work

*corresponding authors

**List of figures:**

**Figure S1. Experimental maps and structural details of *Pha*CCA**

**Figure S2. Comparison of *Pha*CCA structures determined in various conditions**

**Figure S3. SEC-SAXS analysis of *Pha*CCA**

**Figure S4. Nucleotide binding site with CTP / CMPcPP bound or in apo form**

**Figure S5.** **Binding behavior of *Gst*CCA and *Pha*CCA to a tRNA substrate**

**Figure S6. Replacement of flexible loop region does not lead to thermostabilization**

**in *Pha*CCA**

**Figure S7. Analysis of both secondary structure and *in vitro* activity for respective**

**chimeric variants**

**Figure S1. Experimental maps and structural details of *Pha*CCA.** A) N-terminal region of sulfur SAD-phasing structure with associated *2Fo‑Fc* experimental map contoured at 1.2 σ in blue. The sulfur atoms of Met1, Met104 and Met106 are clearly visible in the anomalous difference map contoured at 2.5 σ in orange. B) C-terminal region of sulfur SAD-phasing structure with associated *2Fo‑Fc* experimental map contoured at 1.2 σ in blue. Sulfur atoms of Met204 and Met297 are visible in the anomalous difference map contoured at 2.5 σ in orange. C) D) E) Catalytic site of structure containing CTP, structure obtained in ammonium sulfate and sulfur SAD-phasing structure, respectively, with associated *2Fo‑Fc* maps contoured at 1.2 σ. Polar contacts between SO_4_^2-^ or PO_4_^2-^ and side chains are represented by yellow dashed lines. F) Flexible loop (residues Ser83 to Ser93) in the structure of the complex with CTP and associated *2Fo‑Fc* experimental map contoured at 1.0 σ.

**Figure S2**. **Comparison of *Pha*CCA structures determined in various conditions.** Head, neck, body and tail domains are colored in red, orange, green and blue, respectively. Small molecules visible at the surface of the structures in their solvent shell include *Pha*CCA ligands (CTP, CMPcPP), buffer compounds (acetate, phosphate or sulfate ions) and the cryoprotectant (glycerol). They are represented in ball and stick mode. RMSD values were calculated with Phenix_Compare, using the native structure (A) determined at the highest resolution (1.8 Å, see Table 1 for details) as reference. A) Native structure. B) Native sulfur SAD structure (RMSD = 0.20 Å). C) Structure obtained in the presence of ammonium sulfate (RMSD = 0.22 Å). D) Structure obtained from a crystal soaked in 5 mM CTP (RMSD = 0.16 Å). E) Structure obtained from a crystal soaked in 10 mM CMPcPP (RMSD = 0.19 Å). Structures A, B, D, E were obtained from crystals grown in 100 mM sodium acetate pH 4.5, 1 M di‑ammonium hydrogen phosphate, structure C from a crystal grown in 100 mM sodium acetate pH 5, 3 M ammonium sulfate. F) Superposition of all structures represented in ribbon mode, illustrating the absence of conformational transition between the apo and CTP-bound state.

**Figure S3:** **SEC-SAXS analysis of *Pha*CCA.** A) SEC chromatogram showing a single population. The green and blue windows correspond to SAXS analyses to collect the buffer (180 images) and sample (630 images) signals, respectively. B) Estimate of Rg and I(0) for the 200 first sample images. C) SAXS profile obtained after solvent subtraction by merging individual profiles in the region indicated by the blue bar in B) (images 75-95) where Rg values are stable. D) Guinier plot, associated weighted residual and derived gyration radius (Rg). E) Dimensionless Kratky plot consistent with a compact multidomain protein with a flexible extension [1]. F) Pair atom distance distribution P(r) with derived Rg and Dmax.

**Figure S4. Nucleotide binding site with CTP / CMPcPP bound or in apo form.** A) Complex with CTP, B) with CMPcPP (in two conformations) with the electron density map *2fo-fc* map contoured at 1.3 σ. Residues of the head and neck domains are colored in red and orange, respectively. C) Overlay of *Pha*CCA in apo-form (in grey) and the two complexes highlighting the absence of conformational change and suggesting a key‑and‑lock binding process. The only side chain slightly affected is Arg156. Residues in italic are in direct contact with the ligand (see also **Figure 4**).


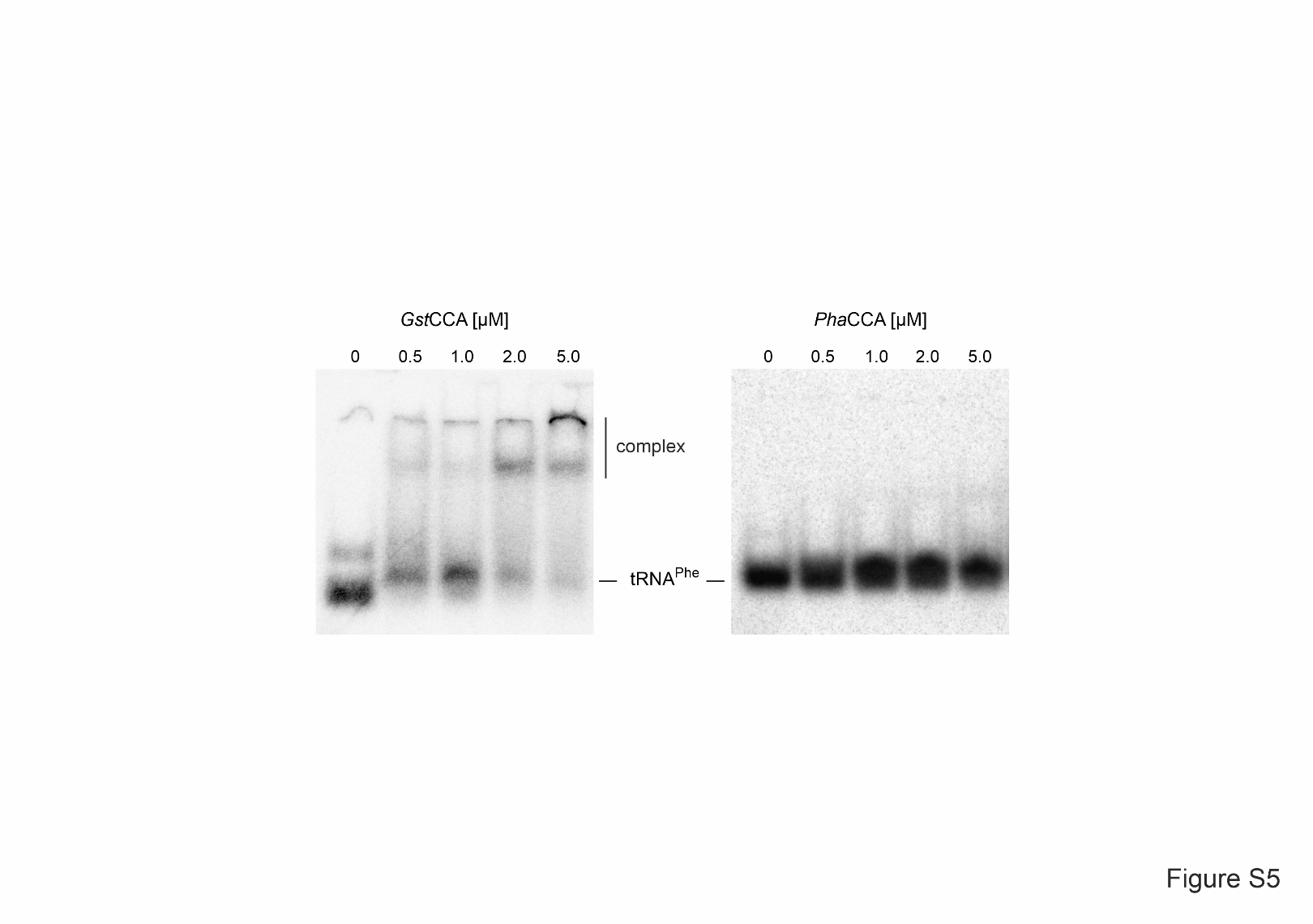


**Figure S5. Binding behavior of *Gst*CCA and *Pha*CCA to a tRNA substrate.** Images of representative electrophoretic mobility shift assays with *Gst*CCA (left) and *Pha*CCA (right). *Gst*CCA exhibits efficient binding to the substrate tRNA^Phe^. After densitometrical quantitation of the radioactive signals, an approximate binding constant of 1.3 µM was determined. In contrast, *Pha*CCA shows no substrate interaction over the whole tRNA concentration range (right). This reduced substrate affinity represents a typical strategy of cold-adapted enzymes to low temperatures.


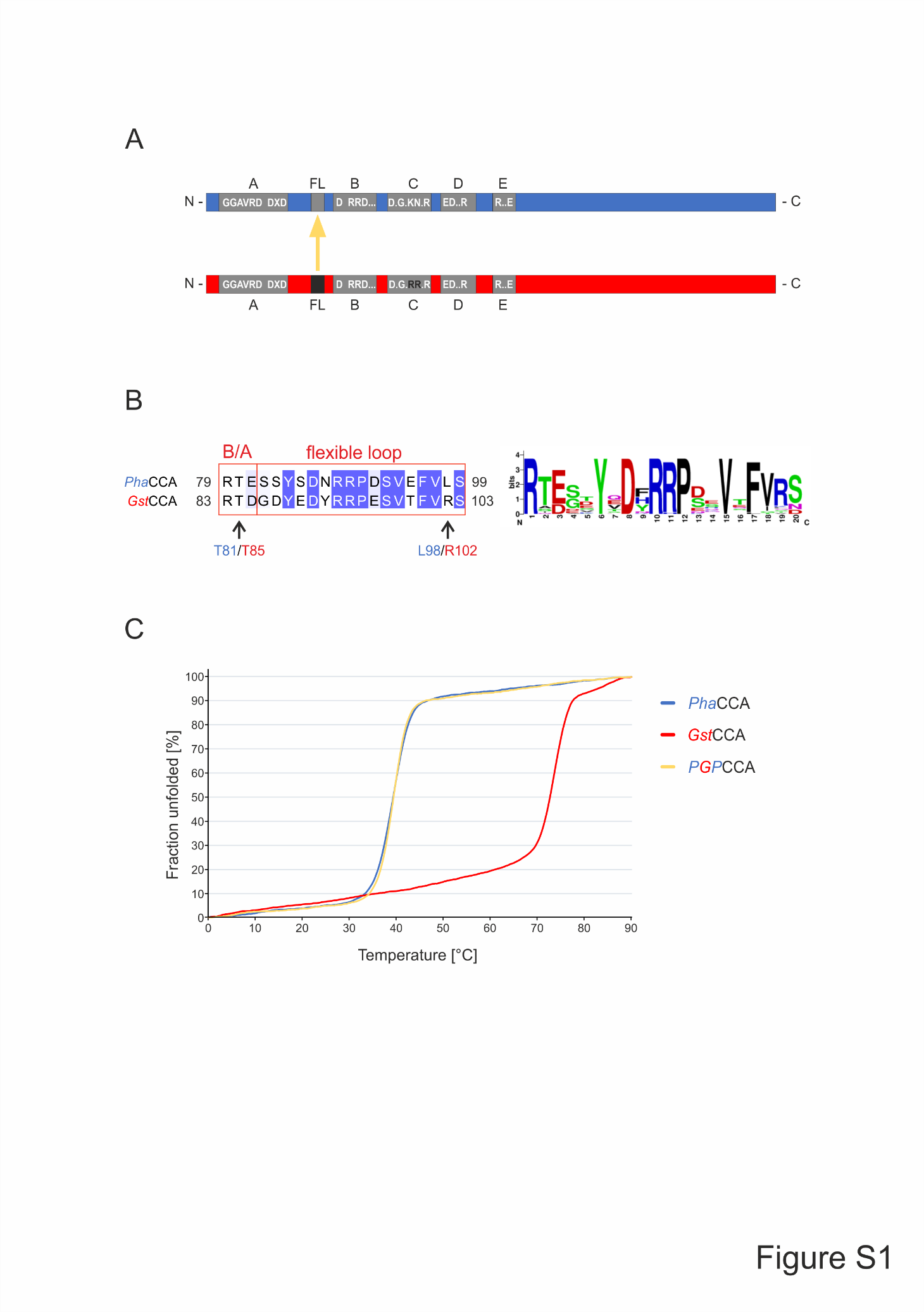


**Figure S6: Replacement of flexible loop region does not lead to thermostabilization in *Pha*CCA.** A) Bar diagrams of *Pha*CCA (blue) and *Gst*CCA (red). Elements of the catalytic core are indicated in grey. Yellow arrow depicts the flexible loop (FL) replacement in *Pha*CCA by the corresponding regions of *Gst*CCA. B) Sequence alignment presenting the fusion positions (T81/T85; L98/R102) of the loop transplantation. On the right, a *Weblogo* [2] serves to illustrate the loop consensus sequence in *Bacillales* [3]. C) Thermal unfolding of CCA-adding enzymes and respective loop chimera; the replacement of the flexible loop by the corresponding *Gst*CCA sequence has no stabilizing effect (yellow).


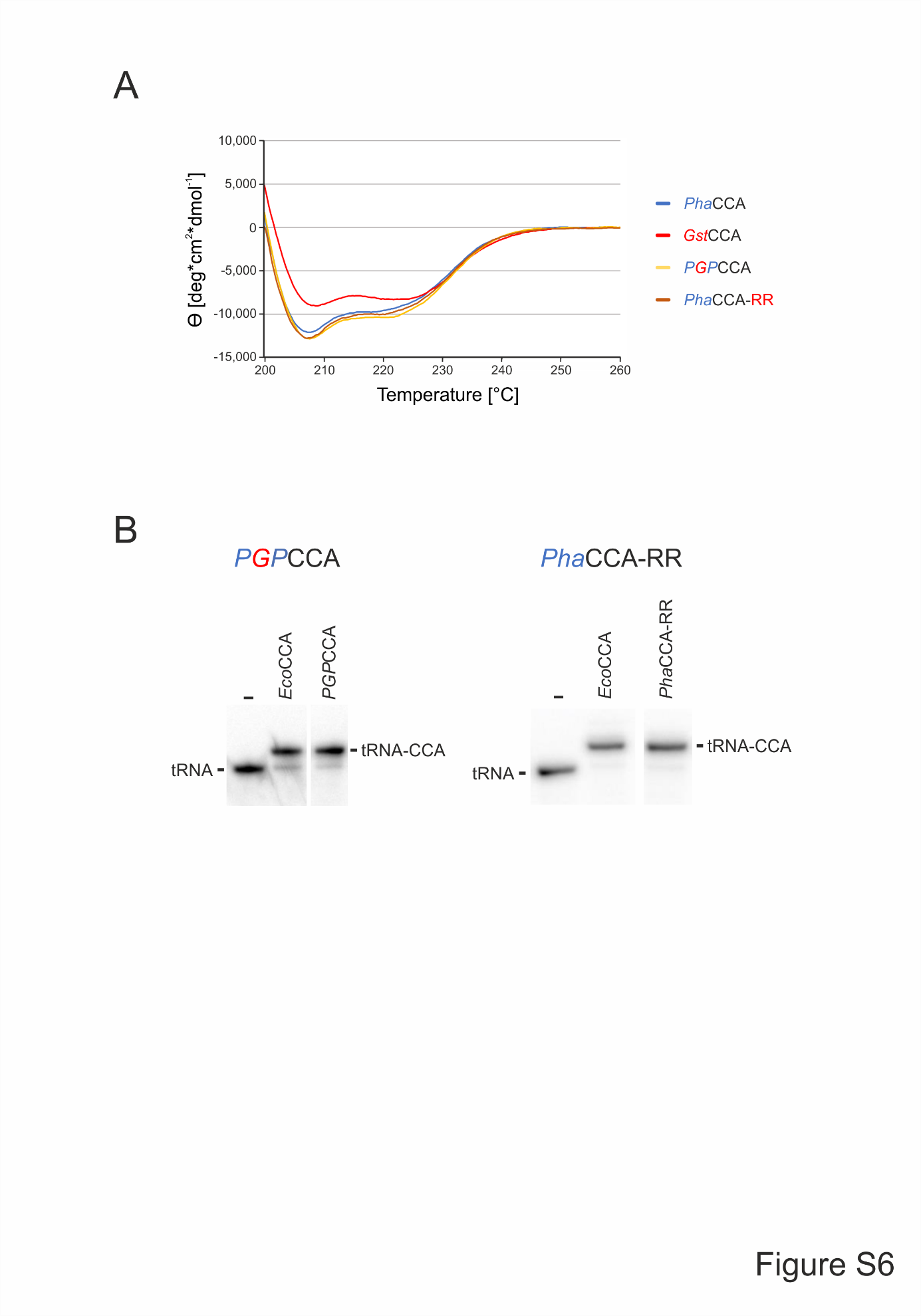


**Figure S7: Analysis of both secondary structure and *in vitro* activity for respective chimeric variants.** A) CD spectral analysis of chimeric variants shows no differing overall secondary structure compared to the parental enzymes. B) As determined by CCA‑incorporation on a radiolabeled yeast tRNA^Phe^ transcript, the investigated chimeric variants are active *in vitro* despite the manipulation within the core motifs; *E. coli* CCA-adding enzyme (*Eco*CCA) served as positive control.

**References**

[1] Receveur-Brechot V, Durand D. How random are intrinsically disordered proteins? A small angle scattering perspective. Curr Protein Pept Sci 2012;13(1):55–75. https://doi.org/10.2174/138920312799277901.

[2] Crooks GE, Hon G, Chandonia J-M, Brenner SE. WebLogo: a sequence logo generator. Genome Res 2004;14(6):1188–90. https://doi.org/10.1101/gr.849004.

[3] Hoffmeier A, Betat H, Bluschke A, Günther R, Junghanns S, Hofmann H-J et al. Unusual evolution of a catalytic core element in CCA-adding enzymes. Nucleic Acids Res. 2010;38(13):4436–47. https://doi.org/10.1093/nar/gkq176.
